# *Campylobacter* infection presenting as pseudo-appendicitis in children: identifying predictors for early diagnosis

**DOI:** 10.3389/fped.2025.1583429

**Published:** 2025-08-18

**Authors:** Michael Schnapper, Yaara Kahan, Amir Klivitsky, Alex Guri, Rachel Shatzman Steuerman, Maya Heled Akiva, Diana Tasher

**Affiliations:** ^1^ Department of Pediatrics, E. Wolfson Medical Center, Holon, Israel; ^2^ Faculty of Medicine, Tel Aviv University, Tel Aviv, Israel; ^3^ Pediatric Infectious Disease Unit, E. Wolfson Medical Center, Holon, Israel; ^4^ Pediatric Infectious Disease Unit, Dana-Dwek Children’s Hospital, Tel Aviv Sourasky Medical Center, Tel Aviv, Israel; ^5^ Division of Pediatrics, Kaplan Medical Center, Rehovot, Israel; ^6^ The School of Medicine, The Hebrew University and Hadassah Medical Center, Jerusalem, Israel; ^7^ Pediatric Infectious Disease Unit, Sheba Medical Center, Tel-Hashomer, Israel; ^8^ Department of Pediatrics, Meir Medical Center, Kfar Saba, Israel

**Keywords:** *Campylobacter*, pseudo-appendicitis, appendicitis, ileocolitis, enteritis, mimicry

## Abstract

**Aims:**

To characterize *Campylobacter* enteritis presenting as pseudo-appendicitis and identify distinguishing predicting factors.

**Methods:**

This retrospective multicentre study included all children <18 years with confirmed *Campylobacter* infection, hospitalized from 2014 to 2023 for presumed appendicitis (pseudo-appendicitis group). Each case was matched with 2 controls with confirmed appendicitis. Multivariable logistic regression analysis was conducted to determine the potential predictors for pseudo-appendicitis.

**Results:**

Fifty-five cases of pseudo-appendicitis were compared with 110 cases of appendicitis. The rate of peritoneal signs was similar between the two groups (78.2% vs. 75.5%, *P* = 0.07). Computed-tomography (CT) scans were performed nearly twice as often in the pseudo-appendicitis group (38% vs. 20%, *P* = 0.01). Broad-spectrum antibiotics were administered to 19 (34.5%) of patients with pseudo-appendicitis and none had surgery. Independent predictors of pseudo-appendicitis included: history of fever (OR: 17.2, 95% CI: 4.7–62.9, *P* < 0.01), WBC <12,000/*μ*l (OR: 9.6, 95% CI: 2.9–31, *P* < 0.01), sonographic signs of enlarged mesenteric lymphadenopathy and/or ileocolitis (OR:5.8, 95% CI:1.8–18.6, *P* = 0.03), no sonographic sign of appendicitis (OR: −5.8, 95% CI: 1.3–25, *P* = 0.02), diarrhea (OR: 3.7, 95% CI:1.2–11.3, *P* = 0.02), and age >14 years (OR:3.3, 95% CI:0.91–12, *P* = 0.06).

**Conclusion:**

The diagnostic challenge of *Campylobacter* pseudo-appendicitis notably led to high rates of CT imaging and unnecessary broad-spectrum antibiotic administration. We identified predictors that may prompt clinicians to consider *Campylobacter* enteritis in selected cases of suspected appendicitis, potentially encouraging early molecular diagnosis and improving patient care.

## Key Notes

• Pseudo-appendicitis is an uncommon presentation of *Campylobacter* enteritis that can be challenging to differentiate from acute appendicitis at onset.
• The frequent use of Computed-Tomography imaging and broad-spectrum antibiotics highlights the need for increased clinical awareness of this presentation.
• We identified potential predictors that may prompt clinicians to consider *Campylobacter* enteritis in selected cases of suspected appendicitis, potentially encouraging early molecular diagnosis and reducing unnecessary treatments, radiation exposure and hospital admissions.

## Introduction

*Campylobacter* enteritis is an important cause of acute diarrhea worldwide. It is typically caused by *Campylobacter jejuni* or *Campylobacter coli* and is largely a foodborne disease. However, *Campylobacter* infection may also be transmitted via water-borne outbreaks and direct contact with animals or animal products (particularly poultry) (1). The clinical presentation typically includes an abrupt onset of abdominal pain and diarrhea, with bloody stools observed in approximately 15%–50% of cases (2).

In rare instances, *campylobacter* infection may mimic other causes of acute abdominal pain, such as acute appendicitis, a phenomenon commonly referred to as pseudo-appendicitis (3, 4). While this phenomenon has been well-documented with *Yersinia enterocolitica* (5–7), cases of *Campylobacter*-associated pseudo-appendicitis in both children and adults have been described only in limited detail (8–10). In addition, most reports involve small case series or individual case reports (11–13), with no direct comparisons to true appendicitis. In cases culminating in surgical intervention, appendicitis was definitively disproved, and even when *Campylobacter spp.* was isolated from the appendix, the organ appeared generally normal without significant inflammation (14).

Severe abdominal pain, pain occurring before the onset of diarrhea, tenderness in the lower quadrants, and absence of diarrhea, are findings that have been reported to delay diagnosis (4, 13, 15). Maintaining a high index of suspicion for *Campylobacter* pseudo-appendicitis cases and performing rapid diagnostic molecular stool testing in selected cases of suspected appendicitis may avoid unnecessary investigations and interventions and hospital stays (16).

The aim of this study was to characterize *Campylobacter* enteritis in children hospitalized with presumed appendicitis and to identify potential predictors that distinguish them from true appendicitis.

## Methods

### Study design

This observational, retrospective case-control study was conducted in four university hospitals located in central Israel, and collected data from January 2014 to December 2020, with one hospital (Wolfson Medical Center) providing additional data from January 2021 to April 2023. Pseudo-appendicitis cases were defined as children under 18 years of age with *Campylobacter* infection who were hospitalized and underwent investigations in suspicion of appendicitis including: a surgical consultation and at least one form of abdominal imaging, such as ultrasound and/or Computed Tomography (CT). Ultrasound and CT imaging interpretations were based on reports by a pediatric radiologist. Sonographic criteria that supported the diagnosis of appendicitis included a non-compressible tubular structure in the right lower quadrant, an appendiceal wall thickness >3 mm, an overall diameter >6 mm, the presence of free fluid in the right lower quadrant, and mesenteric thickening (17).

The presence of *Campylobacter* was confirmed through stool polymerase chain reaction (PCR) and/or stool culture and cases were identified from the microbiological laboratory database.

Stool molecular testing (BioFire FilmArray Gastrointestinal Panel)^1^ (18) was available at all participating centers, with results typically available within 1–2 h. Due to its high cost, stool culture remained the default diagnostic test for evaluating diarrhea, and stool PCR was reserved for selected patients, only upon special request and with approval from an infectious disease specialist, when a rapid diagnosis was expected to influence clinical management.

For patients with pseudo-appendicitis, the Pediatric Appendicitis Score (PAS) was calculated using clinical and laboratory data at presentation. The score ranges from 0 to 10, with established cut-offs defining low risk (<4), indeterminate risk (4–6), and high risk (≥7) for appendicitis (19).

The control group consisted of children aged under 18 years with confirmed appendicitis who underwent appendectomy during the study period, matched by year of hospitalization. Matching was limited to yearly timeframes in order to minimize confounding factors, such as differences in institutional practices, while allowing clinical and demographic variables to be evaluated as potential predictors.

Surgically confirmed appendicitis cases were identified through a combination of International Statistical Classification of Diseases 9 (ICD-9) codes and a departmental surgical and pathologist database.

All pediatric appendicitis cases during the study period were reviewed. For each **case of pseudo-appendicitis**, one case of uncomplicated appendicitis and one case of complicated appendicitis were included. Classification into uncomplicated and complicated appendicitis was based on surgical and histopathology reports. Uncomplicated appendicitis was defined as early-stage appendicitis with an intact appendix wall, while complicated appendicitis was defined by the presence of a perforated appendix, abscess formation, phlegmon, or gangrenous appendicitis (20). Relevant demographic and clinical data were retrieved from the medical records. Patients with missing data for key variables were excluded from the multivariable analysis. Given the expected low proportion of missing data, no data imputation was planned.

The study was approved by the ethics committees of the participating centers (the approval number for the leading center was WOMC-0090-19).

### Statistical analysis

Demographic data, clinical and laboratory results and imaging findings were compared between the study groups. Categorical variables were described as frequency and percentage. Continuous variables were reported as mean and standard deviation or as median and interquartile range (IQR). The Chi-square test and Fisher exact test were used to study the association between categorical variables and the presence of appendicitis while independent sample *T*-test, and Mann–Whitney test were applied to assess the association with continuous variables.

Multivariable logistic regression was used to analyse the association between the potential predictors and the presence of *Campylobacter* pseudo-appendicitis, while controlling for other variables. The CHAID (Chi-square Automatic Interaction Detection) algorithm was selected to identify optimal cutoff values for continuous variables such as age and WBC count, as it enables the identification of multiple data-driven cutoff points. These cut-off values were used to create categorical variables. Consequently, the regression model includes only categorical variables. Variables with a *p*-value <0.01 in the univariate analysis and that were considered most clinically relevant to *Campylobacter* pseudo-appendicitis were selected for inclusion in the multivariable model.

The area under the receiver operating characteristic (ROC) curve was used to evaluate the ability of the regression to discriminate between patients with *Campylobacter* pseudo-appendicitis and patients with appendicitis. All statistical tests were two sided and *P* < 0.05 was considered statistically significant. Statistical analysis used SPSS software (IBM SPSS Statistics for Windows, version 28, IBM Corp., Armonk, NY, USA, 2021).

## Results

### Study population

During the study period, 683 children were diagnosed with *Campylobacter* infection, of whom 55 (8%) were diagnosed with *Campylobacter* pseudo-appendicitis. During the same period 3,318 children were diagnosed with appendicitis, of whom 110 were included as control cases.

### Characteristics of patients with *Campylobacter* pseudo-appendicitis

Characteristics of **patients with** *Campylobacter* Pseudo-appendicitis cases are presented in Table 1.

**Table 1:** Univariate analysis comparing demographic and clinical variables in *Campylobacter* pseudoappendicitis cases and control appendicitis cases.

Variable	*Campylobacter* pseudnoappendicitis (*n* = 55)	Appendicitis (*n* = 110)	*P* Value
Age (years), mean [IQR]	12.7 (10–16)	10.6 (7–14)	0.001
Age >14 years	23 (41.8%)	23 (20.9%)	0.005
Any predisposing illness, *n* (%)	8 (14.5%)	13 (11.8%)	0.62
Male, *n* (%)	33 (60%)	68 (61.8%)	0.82
Interval from onset of symptoms to hospitalization (d), mean [IQR]	2.2 (1–3)	2.1 (1–2.25)	0.012
Peritoneal signs, *n* (%)	43 (78.2%)	83 (75.5%)	0.7
History of diarrhea, *n* (%)	37 (67.3%)	28 (25.5%)	<0.001
History of vomiting, *n* (%)	25 (45.5%)	68 (61.8%)	0.046
Fever prior to admission, *n* (%)	48 (87%)	35 (31.8%)	<0.001
Temperature at time of admission (°C), mean, (range)	37.8 (36.5–40.3)	37.1 (36.5–40.1)	0.001
Laboratory test values, mean [IQR]			
White blood cell count, *10^3^/µl	11.5 (8.3–13.7)	15.9 (12–19)	<0.001
White blood cell count <12,000/μl	36 (65.5%)	25 (22.7%)	<0.001
Absolute neutrophil count, *10^3^/µl	9.1 (6.5–11.4)	13.1 (8.9–16.5)	<0.001
Lymphocyte count, *10^3^/µl	1.4 (0.9–1.7)	1.7 (1–2.1)	0.052
Hemoglobin, g/Dl	13.1 (12.5–14)	12.8 (12.2–13.7)	0.26
Platelet count, *10^3^/µl	208 (172–247)	284 (252–333)	<0.001
C-reactive protein, mg/dl	9.4 (4.5–12.3)	5.9 (0.5–6.5)	<0.001
Imaging findings, *n* (%)			
Abdominal Ultrasound performed	52 (94.5%)	104 (94.5%)	1
Ultrasound findings			
Normal ultrasound	15 (28.8%)	22 (21,1%)	0.28
Ileocolitis	22 (42.3%)	9 (9%)	<0.001
Mesenteric lymphadenitis	22 (42.3%)	14 (14%)	<0.001
Mesenteric lymphadenitis and/or ileocolitis	34 (65.3%)	19 (18.2%)	<0.001
Free Fluid	8 (15.4%)	28 (28%)	0.08
Suspected appendicitis	5 (9.6%)	56 (52.8%)	<0.001
Appendix was visualized	53 (96.3%)	92 (83.6%)	0.02
Abdominal CT scan performed	21 (38.2%)	22 (20%)	0.012
CT findings			
Ileocolitis	17 (80.9%)	2 (9.1%)	<0.001
Mesenteric lymphadenitis	13 (61.9%)	4 (18.2%)	<0.002
Free Fluid	5 (23.8%)	10 (45.5%)	0.13
Suspected appendicitis	0 (0%)	22 (100%)	<0.001

CT, computed tomography.

### Demographic data

The mean age of the patients with *Campylobacter* pseudo-appendicitis was 12.7 years (IQR, 10–16 years) and the majority (60%) were males. There were three patients (0.55%) with comorbidities (obesity, type 1 diabetes mellitus, and asthma).

### Clinical characteristics

The mean duration from abdominal symptom onset to hospital admission was 2.2 days (IQR, 1–3 days). A history of fever was common, reported in 48 cases (87%). Vomiting was described in 25 patients (45.5%). Diarrhea was reported in 37 patients (67.3%), with the majority (32 of 37 patients, 86.4%) describing mild symptoms of 2–3 loose stools a day, without mucus or blood. Notably, 18 of the 55 patients (32.7%) with pseudo-appendicitis had no history of diarrhea. Peritoneal signs, such as abdominal rigidity and rebound tenderness, were documented in the majority of cases (43 patients, 78.2%). The mean Pediatric Appendicitis Score was 4.4 (IQR, 3–6), consistent with an intermediate probability of appendicitis that warrants further diagnostic evaluation.

### Microbiological data

Identification of *Campylobacter* in stool samples was based on culture alone in 45 cases (82%), on both culture and molecular assay (BioFire FilmArray Gastrointestinal Panel) (18) in 7 cases (13%), and on molecular assay alone in 3 cases (5%). Notably, none of the culture positive cases had a negative molecular assay.

The majority of *Campylobacter* isolates were *C. jejuni* (*n* = 39, 71%), followed by *C. coli* (*n* = 7, 13%) and *Campylobacter Spp*. (*n* = 9, 15%).

### Radiological findings

Abdominal ultrasound alone was performed in 34 patients (61.8%), while 19 patients (34.5%) underwent both abdominal ultrasound and CT-scan. CT imaging alone was performed in 2 patients (3.6%). Ultrasound findings were observed in 37 (67.2%) patients including ileocolitis (wall thickening of the ileum and/or colon), mesenteric lymphadenitis, free fluid and suspicion of appendicitis, the later was reported in only 5 cases (9.6%). Seven patients with normal ultrasound had abnormal CT scans showing ileocolitis. In all cases CT-scan excluded appendicitis.

### Management of cases with *Campylobacter* pseudo-appendicitis before diagnosis

Twenty-two patients (40%) were prescribed broad spectrum antibiotic for suspected intra-abdominal infections, a combination beta-lactam, and metronidazole with/without aminoglycoside. None of the cases of *Campylobacter* pseudo-appendicitis underwent surgery.

### Comparative findings between *Campylobacter* pseudo-appendicitis and acute appendicitis, including simple and complicated appendicitis

Table 1 presents the univariate analysis comparing demographic and clinical variables between patients with *Campylobacter* pseudo-appendicitis and all control patients with appendicitis. Table 2 provides further comparisons between pseudo-appendicitis, simple appendicitis, and complicated appendicitis groups.

**Table 2:** Univariate analysis comparing demographic and clinical variables in *Campylobacter* pseudoappendicitis cases and control simple and complicated appendicitis cases.

Variable	*Campylobacter* pseudnoappendicitis (*n* = 55)	Simple appendicitis (*n* = 55)	*P* value Pseudo.* vs. Simple appendicitis	Complicated Appendicitis (*n* = 55)	*P* value Pseudo.* vs. Complicated appendicitis
Age (years), mean [IQR]	12.7 (10–16)	11 (9–14)	**0.034**	9.8 (6–14)	**0.001**
Age >14 years	23 (41.8%)	12 (21.8%)	**0.024**	11 (20%)	**0.02**
Any predisposing illness, *n* (%)	8 (14.5%)	5 (9%)	0.55	8 (14.5%)	1
Male, *n* (%)	33 (60%)	31 (56%)	0.7	18 (33%)	**0.004**
Interval from onset of abdominal symptoms to hospitalization (d), mean [IQR]	2.2 (1–3)	1 (0–1)	**0.001**	2.4 (1–3)	0.9
Peritoneal signs, *n* (%)	43 (78.2%)	38 (69%)	0.28	45 (81.8%)	0.8
History of diarrhea, *n* (%)	37 (67.3%)	9 (16.3%)	**<0.001**	19 (34.5%)	**0.001**
History of vomiting, *n* (%)	25 (45.5%)	26 (47.2%)	0.85	41 (74.5%)	**0.003**
Fever prior to admission, *n* (%)	48 (87%)	7 (12.7%)	**<0.001**	25 (45.5%)	**<0.001**
Temperature at time of admission (°C), mean, (range)	37.8 (36.5–40.3)	37.1 (36.1–38.7)	**<0.001**	37.3 (36.3–40.1)	**0.001**
Laboratory test values, mean [IQR]					
White blood cell count, *10^3^/µl	11.5 (8.3–13.7)	15 (11.1–17.4)	**0.001**	17.4 (13.7–19.8)	**<0.001**
White blood cell count <12,000/μl	36 (65.5%)	17 (30%)	**<0.001**	8 (14.5%)	**<0.001**
Absolute neutrophil count, *10^3^/µl	9.1 (6.5–11.4)	12 (7.5–14.7)	**0.001**	14.5 (11–17)	**<0.001**
Lymphocyte count, *10^3^/µl	1.4 (0.9–1.7)	2 (1.2–2.2)	**0.002**	1.5 (1.3–1.9)	0.66
Hemoglobin, g/Dl	13.1 (12.5–14)	13 (12.2–13.8)	0.5	12.8 (12.1–13.3)	0.8
Platelet count, *10^3^/µl	208 (172–247)	295 (256–314)	**<0.001**	297 (245–347)	**<0.001**
C-reactive protein, mg/dl	9.4 (4.5–12.3)	1 (0.11–1.98)	**<0.001**	10.4 (2.3–17.5)	0.42
Imaging findings, *n* (%)					
Abdominal ultrasound performed	52 (94.5%)	52 (94.5%)	0.24	51 (89%)	0.48
Ultrasound findings					
Normal ultrasound	15 (28.8%)	17 (32.6%)	0.67	5 (9.8%)	**0.013**
Ileocolitis	22 (42.3%)	2 (3.8%)	**<0.001**	7 (13.7%)	**0.002**
Mesenteric lymphadenitis	22 (42.3%)	8 (15.3%)	**0.004**	6 (11.7%)	**<0.001**
Mesenteric lymphadenitis and/or ileocolitis	34 (65.3%)	8 (14.5%)	**<0.001**	11 (21.5%)	**<0.001**
Free fluid	8 (15.4%)	4 (7.2%)	0.22	24 (47%)	**0.001**
Suspected appendicitis	5 (9.6%)	30 (54.5%)	**<0.001**	39 (76.4%)	**<0.001**
Appendix was visualized	50 (96.1%)	37 (71.1%)	**0.001**	37 (72.5%)	**0.001**
Abdominal CT scan performed	21 (38.2%)	7 (12.7%)	**0.002**	15 (27.3%)	0.3
CT findings					
Ileocolitis	17 (80.9%)	0 (0%)	**<0.001**	2 (13.3%)	**<0.001**
Mesenteric lymphadenitis	13 (61.9%)	0 (0%)	**0.007**	4 (26.7%)	**0.04**
Free Fluid	5 (23.8%)	4 (57.1%)	0.16	6 (40%)	0.46
Suspected appendicitis	0 (0%)	7 (100%)	**<0.001**	16 (100%)	**<0.001**

Bold values indicate statistical significance (*P* < 0.05).

CT, computed tomography; Pseudo, pseudoappendicitis.

The mean duration of abdominal symptoms before admission was longer in the pseudo-appendicitis group compared to the simple appendicitis group (2.2 vs. 1 day, *P* = 0.001), but similar to the complicated appendicitis group (2.2 vs. 2.4 days, *P* = 0.9). The rate of peritoneal signs on physical examination was similar in patients with *Campylobacter* pseudo-appendicitis compared to patients with appendicitis (78.2% vs. 75.5%, *P* = 0.07). However, patients with *Campylobacter* pseudo-appendicitis more frequently presented with fever (87% vs. 31.8%, *P* < 0.001) and diarrhea (67.3% vs. 25.5%, *P* < 0.001), while vomiting was less common (45.5% vs. 61.8%, *P* = 0.046). Laboratory findings revealed distinct patterns. The *Campylobacter* group demonstrated significantly lower WBC (11.5 vs. 15.9 × 10^3^/µl, *P* < 0.001), lower absolute neutrophil counts (9.1 vs. 13.1 × 10^3^/µl, *P* < 0.001), lower platelet counts (208 vs. 284 × 10^3^/µl, *P* < 0.001), but higher C-reactive protein levels (9.4 vs. 5.9 mg/dl, *P* < 0.001).

### Potential predictors associated with *Campylobacter* pseudo-appendicitis

A total of 8 patients (fewer than 5% of the cohort) were excluded from the multivariable analysis because ultrasound examinations were not performed.

**Figure 1:**
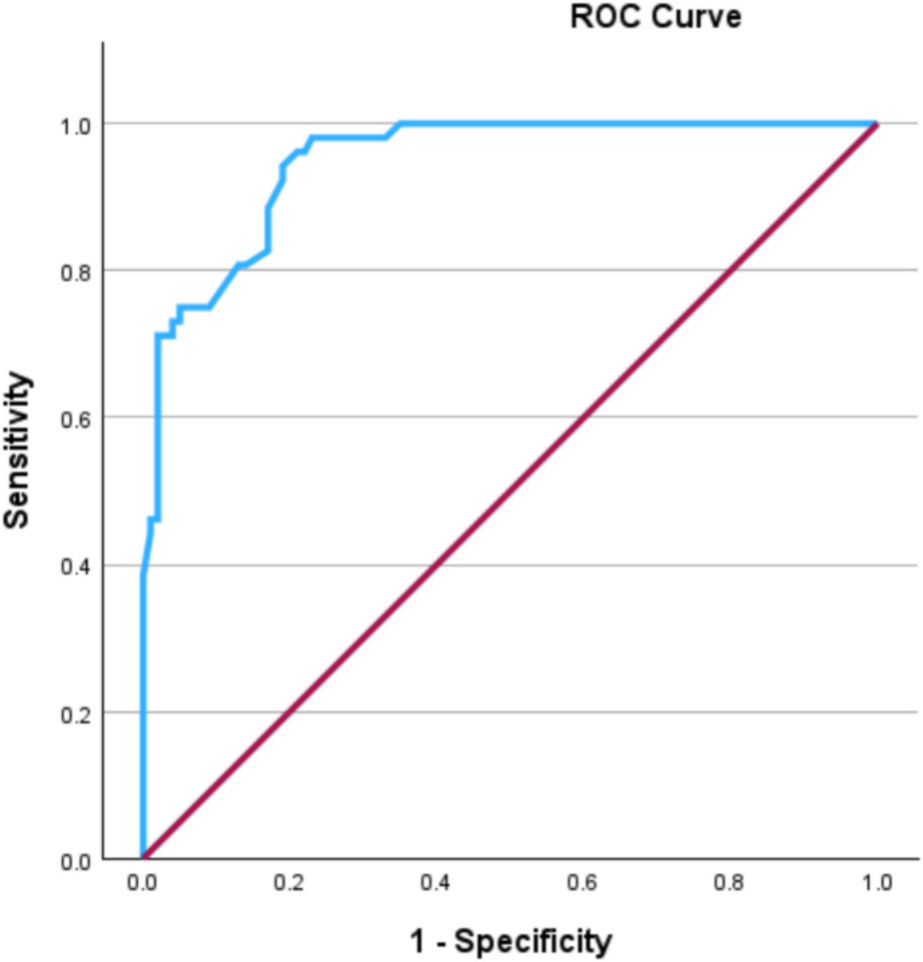
Receiver operating characteristic (ROC) curve demonstrating the discrimination ability of the model to predict *Campylobacter* Pseudo-appendicitis.

Using a logistic multivariable regression analysis, we adjusted for the following variables, which were identified as independently associated with *Campylobacter* pseudo-appendicitis compared to confirmed appendicitis: history of fever (OR: 17.2, 95% CI: 4.7–62.9, *P* < 0.001), WBC < 12,000/μl (OR: 9.6, 95% CI: 2.9–31, *P* < 0.001), sonographic findings of enlarged mesenteric lymph nodes and/or ileocolitis (OR: 5.8, 95% CI: 1.8–18.6, *P* = 0.03), absence of sonographic evidence of suspected appendicitis (OR: −5.8, 95% CI: 1.3–25, *P* = 0.02), history of diarrhea (OR: 3.7, 95% CI: 1.2–11.3, *P* = 0.02), and age >14 years (OR: 3.3, 95% CI: 0.91–12, *P* = 0.06) (Table 2). The same variables remained significant predictors when *Campylobacter* pseudo-appendicitis was compared to uncomplicated and complicated appendicitis separately. The area under the curve of the ROC of the final prediction model was 0.95 (95% CI: 0.92–0.98) (Figure 1). The optimal cutoff value was identified using the maximum Youden's Index (15.8%), which yielded a specificity of 97% and a sensitivity of 96.2%.

## Discussion

In our study we provide a detailed comparison between patients with *Campylobacter* pseudo-appendicitis and confirmed acute appendicitis. High rates of peritoneal signs with lack of predominant diarrhea in both groups illustrate the diagnostic challenges, as evident by the high rate of CT imaging and administration of broad-spectrum antibiotics. We identified a number of predictors that should alert clinicians to consider *Campylobacter* infection in the differential diagnosis of acute appendicitis. These predictors ranked by strength of association include: history of fever; WBC count below 12,000/µl; sonographic findings of enlarged mesenteric lymph nodes and/or ileitis/colitis; no sonographic signs of appendicitis; history of diarrhea; and age above 14 years.

Our results indicated comparable history and physical examination findings for *Campylobacter* pseudo-appendicitis and appendicitis, with a similar interval from symptom onset to hospitalization and similar rates of localized pain with peritoneal signs. Diarrhea, albeit mostly mild, and fever were often present on presentation in *Campylobacter* cases, and therefore may serve as diagnostic clues. Unsurprisingly, fever was the strongest suggestive sign in *Campylobacter* cases while diarrhea was unexpectedly absent in about 33% of cases. These findings are consistent with previous literature of *Campylobacter* pseudo-appendicitis (10, 13, 20) and emphasize the importance of thoroughly investigating any preceding history of diarrhea and fever, even if not prominent at presentation, as this may suggest an alternative diagnosis.

We observed a distinctive blood count profile in *Campylobacter* pseudo-appendicitis cases, where the WBC count, absolute neutrophil count (ANC), and platelet count were lower than those seen in patients with appendicitis. These findings align with previous studies that reported normal or minimally elevated WBC counts in *Campylobacter* infectionns (20, 21).

In contrast, we observed higher CRP (C-reactive protein) levels in the *Campylobacter* group compared to cases with simple appendicitis, but not compared to those with complicated appendicitis. Although this difference was not statistically significant in the multivariable analysis, given that CRP is a late-phase inflammatory marker, these findings may reflect the longer duration of illness before admission among *Campylobacter* patients and those with complicated appendicitis compared to simple appendicitis (22). A recent study that similarly noted significantly higher CRP levels in all cases of *Campylobacter* gastroenteritis compared to patients with appendicitis. However, this study did not specifically address CRP levels in patients with *Campylobacter* pseudo-appendicitis (17).

Interestingly, older children (age >14 years) was predictive of *Campylobacter* pseudo-appendicitis. This is in accordance with limited evidence from previous reports. Worldwide studies have concluded that the age distribution of *Campylobacter* enteritis in industrialized countries is bimodal, with the first peak occurring in early childhood and the second peak in young adulthood (23, 24). Immunity, anatomical and physiological characteristics of the gastrointestinal system, and environmental exposure are likely to influence the age-related rates of *Campylobacter* infection and the predilection for specific manifestations such as pseudo-appendicitis (23).

Ultrasonography results from the *Campylobacter* pseudo-appendicitis group, revealed mesenteric adenitis and/or ileitocolitis in the majority of cases, suggesting the presence of bacterial colitis (3). However, clinical uncertainty led to CT imaging being performed almost twice as frequently in the *campylobacter* group vs. the appendicitis control group (38% vs. 20%, *P* = 0.01). The inappropriate use of antibiotics was common, with broad-spectrum antibiotics often prescribed empirically before the microbiological diagnosis of *Campylobacter* was confirmed.

CT imaging, which unfortunately exposes children to ionizing radiation, was highly accurate in excluding appendicitis in our study. Accordingly, none of the patients with *Campylobacter* pseudo-appendicitis underwent surgery. This may be attributed to both the accuracy of CT imaging and the growing trend towards non-operative management of appendicitis in children, which tends to promote a more conservative approach in cases of suspected appendicitis (24–28).

The use of stool culture for emergency care decision-making in diagnostically challenging cases is not practical due to its prolonged turnaround time. However, stool molecular diagnostic methods can rapidly establish a diagnosis of *Campylobacter* colitis with even higher sensitivity than traditional culture methods, which may miss up to 28% of true-positive cases (29). Despite the availability of stool PCR testing at all participating centers, the utilization in our study population was low (18%). This probably reflects both limited awareness of this unusual *Campylobacter* presentation among healthcare providers and the practical challenges of obtaining stool samples. These challenges include the absence of diarrhea at admission or the need to wait for patients to produce a stool sample. Notably, recent studies have demonstrated that rectal swabs perform as well as conventional stool samples for the molecular detection of gastrointestinal pathogens, though no tests are FDA-cleared for this use (30–33).

Using rectal swabs for point-of-care testing in a selective group of children with suspected appendicitis and clinical characteristics suggestive of *Campylobacter* infection based on potential predictors may facilitate faster diagnosis, reduce unnecessary imaging and radiation exposure, minimize antibiotic use, and improve patient care, including shortening the length of hospitalization (30–32).

To our knowledge, this is the largest reported cohort of patients with *Campylobacter* enteritis presenting with presumed appendicitis to date. In addition, the inclusion of patients who underwent both surgical consultation and imaging studies, along with the high percentage of CT imaging in our cohort, suggest that we captured cases with significant diagnostic uncertainty, rather than just typical *Campylobacter* gastroenteritis. Furthermore, the control group included both uncomplicated and complicated cases, thereby ensuring a broad representation of appendicitis.

However, our study has certain limitations. Its retrospective design introduces potential biases, including the subjective interpretation of physical examinations and sonographic findings. Additionally, our study relied on radiologist reports of ultrasound images rather than point-of-care ultrasound (POCUS), and therefore may not reflect real-time decision-making in the emergency setting. The availability of both stool PCR testing and radiologist-performed ultrasound in the participating medical centers may limit the generalizability of our findings to resource-limited settings.

By inclusion of only patients with confirmed *Campylobacter* infection, we potentially missed cases where clinical suspicion was low and stool analyses were not requested. Such a situation could occur if appendicitis was excluded by imaging or surgery, or following spontaneous recovery. Thus, underestimating the true prevalence of *Campylobacter* pseudo-appendicitis.

Furthermore, our study did not include a comparative analysis with patients diagnosed with typical *Campylobacter* gastroenteritis. In future studies, this perspective may elucidate more distinguishing clinical features of pseudo-appendicitis.

The inclusion of only surgically confirmed appendicitis cases in the control group limits the generalizability of our findings to non-operatively managed appendicitis patients. A further limitation of this study is the relatively small sample size. Lastly, building models to predict rare situations, such as *Campylobacter* pseudo-appendicitis, is inherently challenging. Alternatively, we propose a set of independent predictive characteristics that may aid clinicians to increase their index of suspicion for *Campylobacter* pseudo-appendicitis in cases of suspected appendicitis.

Larger prospective studies are needed to validate a scoring system for differentiating *Campylobacter* pseudo-appendicitis from appendicitis in clinical practice. These studies should also include non-operatively managed appendicitis and investigate whether, in selected cases, the use of point-of-care ultrasound and rapid stool molecular assays in the emergency department setting can help prevent unnecessary imaging and hospitalizations.

In conclusion, our results indicate that pseudo-appendicitis is an uncommon presentation of *Campylobacter* enteritis that can be challenging to differentiate from acute appendicitis at onset. The frequent use of CT imaging and administration of broad-spectrum antibiotics, highlights the need for increased clinical awareness of this entity. We suggest that this may be resolved by considering the most suggestive predictors, ranked by significance, as described above. These observations may contribute to the early diagnosis of *Campylobacter* pseudo-appendicitis by molecular testing, potentially reducing unnecessary antibiotic treatments, radiation exposure and hospital admissions.

## Data Availability

The raw data supporting the conclusions of this article will be made available by the authors, without undue reservation.

## Footnotes

^1^bioMérieux. Available online at: https://www.biomerieux.com/us/en.html (Accessed July 19, 2025).
